# Urothelial ATP exocytosis: regulation of bladder compliance in the urine storage phase

**DOI:** 10.1038/srep29761

**Published:** 2016-07-14

**Authors:** Hiroshi Nakagomi, Mitsuharu Yoshiyama, Tsutomu Mochizuki, Tatsuya Miyamoto, Ryohei Komatsu, Yoshio Imura, Yosuke Morizawa, Miki Hiasa, Takaaki Miyaji, Satoru Kira, Isao Araki, Kayoko Fujishita, Keisuke Shibata, Eiji Shigetomi, Youichi Shinozaki, Reiko Ichikawa, Hisayuki Uneyama, Ken Iwatsuki, Masatoshi Nomura, William C. de Groat, Yoshinori Moriyama, Masayuki Takeda, Schuichi Koizumi

**Affiliations:** 1 Department of Urology, Interdisciplinary Graduate School of Medicine, University of Yamanashi, Yamanashi 409-3898, Japan; 2 Department of Neuropharmacology, Interdisciplinary Graduate School of Medicine, University of Yamanashi, Yamanashi 409-3898, Japan; 3 Department of Membrane Biochemistry, Okayama University Graduate School of Medicine, Density, and Pharmaceutical Science, Okayama 700-8530, Japan; 4 Advanced Science Research Center, Okayama University, Okayama 700-8530, Japan; 5 Department of Urology, Shiga University of Medical Science, Shiga 520-2192, Japan; 6 Japan Science and Technology Agency, CREST, Tokyo 102-0076, Japan; 7 Institute of Life Sciences, Ajinomoto Co. Inc., Kawasaki 210-8681, Japan; 8 Department of Endocrine and Metabolic Diseases/Diabetes Mellitus Kyushu University Hospital, Fukuoka 812-8582, Japan; 9 Department of Pharmacology and Chemical Biology, University of Pittsburgh School of Medicine, Pittsburgh, PA 15213, USA

## Abstract

The bladder urothelium is more than just a barrier. When the bladder is distended, the urothelium functions as a sensor to initiate the voiding reflex, during which it releases ATP via multiple mechanisms. However, the mechanisms underlying this ATP release in response to the various stretch stimuli caused by bladder filling remain largely unknown. Therefore, the aim of this study was to elucidate these mechanisms. By comparing vesicular nucleotide transporter (VNUT)-deficient and wild-type male mice, we showed that ATP has a crucial role in urine storage through exocytosis via a VNUT-dependent mechanism. VNUT was abundantly expressed in the bladder urothelium, and when the urothelium was weakly stimulated (i.e. in the early filling stages), it released ATP by exocytosis. VNUT-deficient mice showed reduced bladder compliance from the early storage phase and displayed frequent urination in inappropriate places without a change in voiding function. We conclude that urothelial, VNUT-dependent ATP exocytosis is involved in urine storage mechanisms that promote the relaxation of the bladder during the early stages of filling.

The urinary bladder has two main functions. One is the collection of urine; the other is expulsion of urine at the appropriate time and place^1^. The impairment of bladder compliance (BCP), defined as the change in volume per unit change of pressure during bladder filling^2^, leads to decreased bladder capacity and increased intravesical pressure in the storage phase, resulting in the failure of these functions. BCP is therefore an important indicator of the storage capacity of the bladder. BCP is variably dependent upon multiple factors. The bladder wall consists of collagen, elastin and smooth muscle, in addition to nerves, blood vessels and epithelium, and the interrelationships between these elements determines BCP^3^. The epithelial lining of the urinary bladder, the bladder urothelium, is classically believed to be a passive barrier^4^, playing a pivotal role as an interface^5^,^6^,^7^. The bladder urothelium senses and responds to various chemical, mechanical and thermal stimuli, releasing chemical factors such as ATP^8^,^9^. Urothelial ATP has important roles in regulating normal urinary bladder function. Acting on P2 purinoceptors on suburothelial sensory afferent nerve fibres, it signals urinary bladder filling^10^,^11^. Additionally, bladder filling-induced urothelial ATP acting on umbrella P2 receptors (apical uroepithelial cells) as an autocrine signal increases the mucosal surface area by exocytosis and the fusion of a subapical pool of fusiform/discoidal-shaped vesicles (FDVs) with the apical membrane of the superficial umbrella cells^12^. Other transmitters, such as acetylcholine^13^ and cytokines^14^, are released from the urothelium via ATP stimulation. We have previously shown that cultured urothelial cells release ATP in response to mechanical stretch stimulation^15^. However, the mechanisms underlying urothelial ATP release during bladder filling remain unclear. ATP is released via connexin hemichannels^16^, pannexin^17^, several anion channels^18^,^19^, P2 × 7 receptors^20^,^21^ and the cystic fibrosis transmembrane conductance regulator^22^. ATP can also be released by exocytosis^23^. Solute carrier family 17 [vesicular nucleotide transporter (VNUT)], member 9 (*SLC17A9*), a novel member of an anion transporter family, has recently been identified^24^. VNUT is responsible for the exocytosis of ATP and is widely expressed in various human and murine tissues^24^, including epithelial cells^25^,^26^. In the present study, we focused on VNUT in the bladder to clarify the mechanisms that underlie urothelial ATP release, along with the physiological consequences of this. We show that VNUT is present and functional in the bladder urothelium and that it plays an essential role in the exocytosis of ATP during stimulation by weak stretch. VNUT-deficient (VNUT-KO) mice show a decrease in BCP accompanied by frequent urination behaviour. Urothelial VNUT-dependent ATP exocytosis has an important role in urine storage mechanisms that promote bladder relaxation during the early stages of filling.

## Results

### VNUT was highly expressed in bladder epithelium

**Figure 1:**
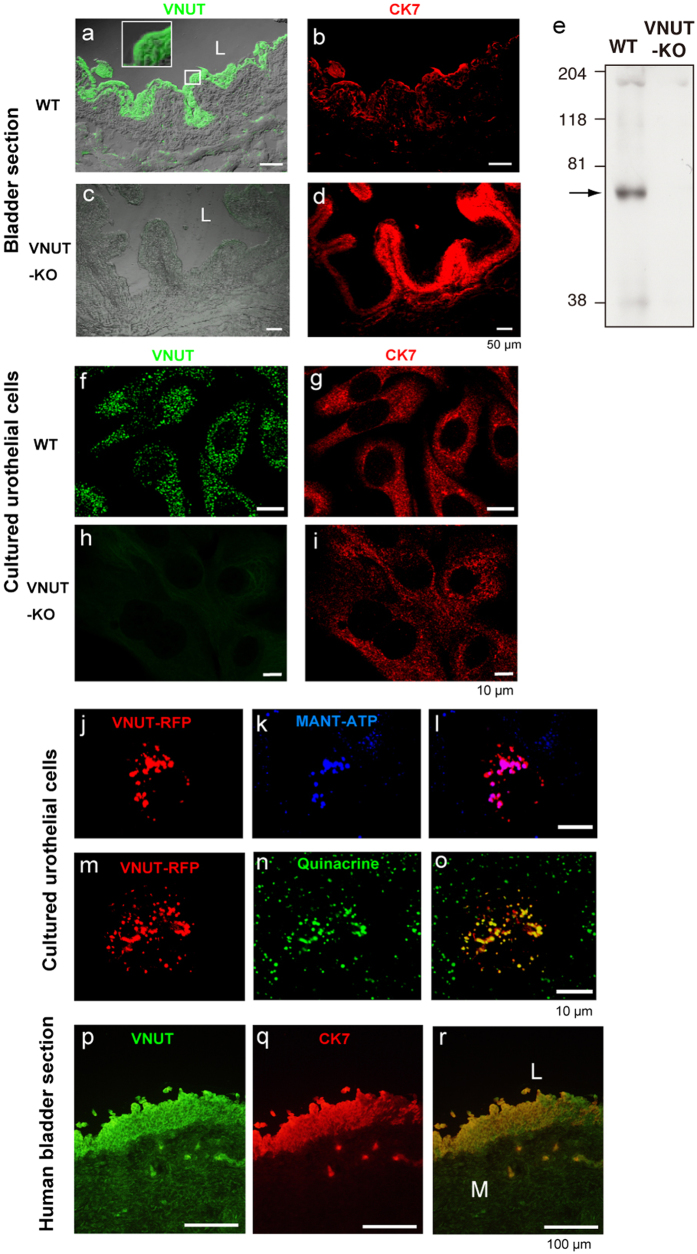
VNUT-positive signals in the bladder and cultured urothelial cells. (**a–d**) Immunohistochemical analysis of VNUT ((**a**,**c**) in green) and CK7 (**b,d**), in red) in mouse whole bladder tissue merged with a phase-contrast image. Images (**a,b**) show tissue from WT mice, while (**c,d**) show tissue from VNUT-KO mice. The magnified inset in (**a**) shows VNUT localization in each urothelial cell layer. L, lumen; scale bars, 50 μm. (**e**) Western blot of VNUT-positive signals in the bladder. The membranes of bladder (50 μg) prepared from WT and VNUT-KO mice were analysed by western blot with anti-VNUT-antibody. VNUT-positive signal (arrow) was not detected in the bladder obtained from VNUT-KO mice. (**f–i**) Immunocytochemical analysis of VNUT (**f,h**), in green) and CK7 (**g,i**), in red) in cultured urothelial cells. Images (**f,g**) show urothelial cells obtained from WT mice, while (**h,i**) show cells from VNUT-KO mice. Scale bars, 10 μm. (**j–l**) Co-localization of VNUT-RFP with MANT-ATP in mouse primary urothelial cells. Cultured urothelial cells were transfected with VNUT-RFP, followed by staining with MANT-ATP, a fluorescent ATP. (**j**) Labelling of urothelial cells with VNUT-RFP (red). (**k**) Urothelial cells incubated with MNAT-ATP (blue). (**l**) Merged image, showing VNUT-RFP localized with vesicles where MANT-ATP accumulated. (**m–o**) Co-localization of VNUT-RFP with quinacrine. Urothelial cells were transfected with VNUT-RFP followed by staining with quinacrine. (**m**) Labelling of urothelial cells with VNUT-RFP (red). (**n**) Labelling of urothelial cells with quinacrine (green). (**o**) Merged image showing VNUT-RFP co-localised with quinacrine-positive signals. Scale bars, 10 μm. (**p–r**) Immunohistochemical analyses of the human bladder. (**p**) VNUT-positive staining (green) was localized in each urothelial cell layer. (**q**) Urothelial marker CK7-positive staining (red) in human bladder tissue. (**r**) Merged image of (**p,q**). Scale bars, 100 μm.

VNUT immunoreactivity (IR) was broadly co-expressed with cytokeratin-7 (CK7), an intermediate filament protein that is present in all urothelial layers. VNUT IR was weak in the detrusor muscle and in the suburothelial space of the bladders of wild-type (WT) mice (Fig. 1a,b). VNUT IR was absent in the bladders of VNUT-KO mice (Fig. 1c,d). These findings were confirmed using western blot analysis (Fig. 1e). Haematoxylin and eosin (H&E) staining of the bladders of VNUT-KO mice did not reveal any gross abnormalities (data not shown). The immunocytochemical analysis of primary urothelial cells also showed VNUT IR in CK7-positive cells of WT mice (Fig. 1f,g) but not in those of VNUT-KO mice (Fig. 1h,i). VNUT IR was associated with vesicular-like structures (Fig. 1f).

To investigate whether VNUT-positive vesicles contained ATP, we visualised VNUT and ATP using VNUT-red fluorescent protein (RFP) and the fluorescent ATP analogue MANT-ATP, respectively. MANT-ATP (Fig. 1k) accumulated in VNUT-RFP-positive vesicles (Fig. 1j,l). VNUT-RFP-positive signals were not observed in all of the cells because of the low transfection efficiency. However, almost all VNUT-RFP-positive vesicles contained ATP within the vesicles. We also stained urothelial cells with VNUT-RFP (Fig. 1m) and quinacrine, an acridine compound that stains acidic vesicles (Fig. 1n). We observed that the VNUT-RFP-positive signals co-localised with quinacrine (Fig. 1o). To visualize the dynamics of exocytosis for ATP-containing vesicles during mechanical stimulation, we stimulated quinacrine-stained urothelial cells with a micropipette (Fig. S1a). The intensity of staining in individual quinacrine vesicles faded or disappeared with different kinetics in response to this stimulus. Supplementary Fig. S1 shows quinacrine-positive vesicles before and 80 s after mechanical stimulation (Fig. S1b,c, respectively).

An immunohistochemical analysis of human bladder tissues also showed strong VNUT protein expression in the urothelial cell layers but considerably less expression in the detrusor muscle suburothelial spaces (Fig. 1p). These VNUT-positive immunohistochemical signals co-localized with CK7-positive signals (Fig. 1q,r).

### Urothelial cells released ATP in response to mechanical stretching

**Figure 2:**
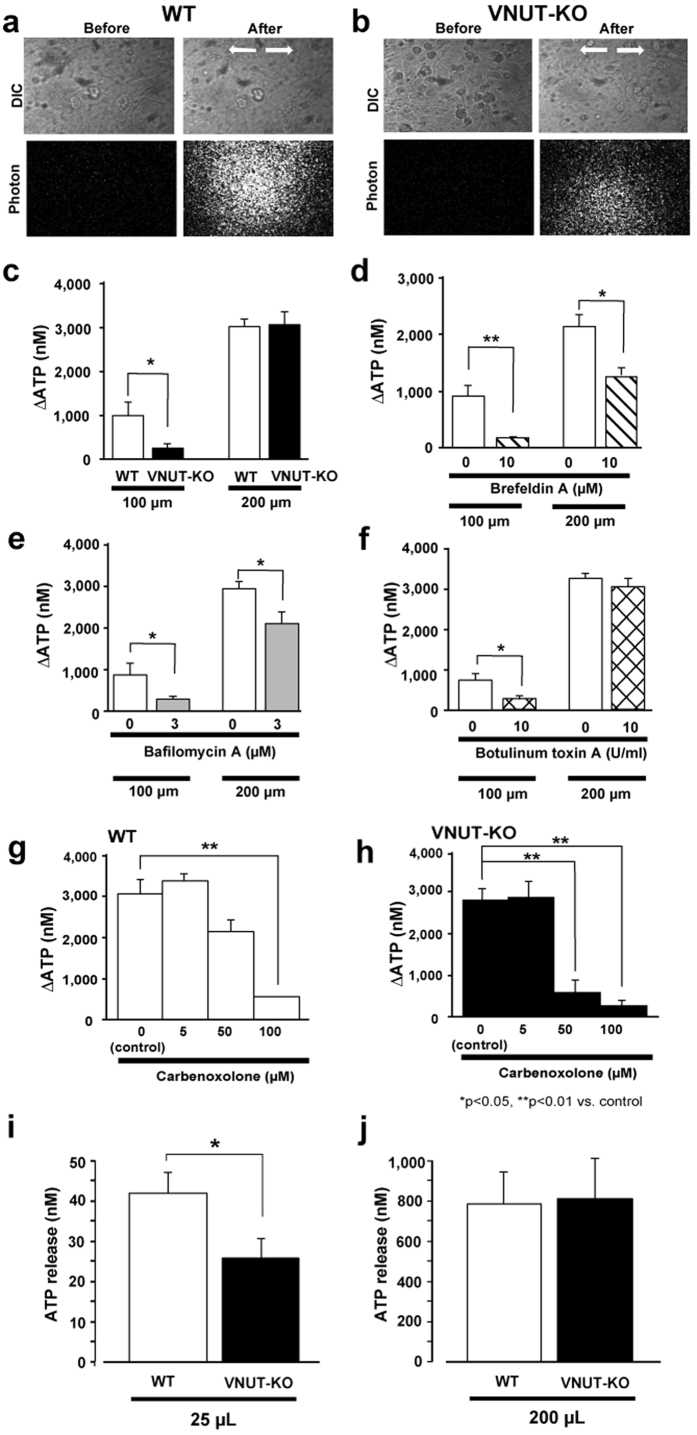
Visualization and characterization of stretch-evoked ATP release from primary urothelial cell cultures or the bladder *in vivo*. (**a,b**) The upper panels show urothelial cells in differential interference contrast images (DIC) for WT and VNUT-KO samples, and the lower panels show photon counting images (white dots) in the corresponding fields. The stretch speed was 100 μm/s, and the distance was 100 μm (10% elongation). Cells were stretched transversely (as indicated by the arrows). (**c**) The average amount of ATP released from urothelial cells in response to a 100 μm (10%) and a 200 μm (20%) stretch in WT (n = 10) and VNUT-KO cells (n = 8). Effects of (**d**) Brefeldin A (n = 4, control n = 4), (**e**) bafilomycin A (n = 8, control n = 8) and (**f**) botulinum toxin A (n = 6, control = 7) on ATP release with stretching (10% or 20%) in WT cells. The average amount of ATP released from urothelial cells that were obtained from WT (**g**) and VNUT-KO (**h**) mice in response to 200 μm of stretching (20% elongation). The effects of various concentrations of carbenoxolone (5–100 μM, n = 4–6 at each concentration) are shown. (**i,j**) The saline infusion-evoked ATP release from the mouse bladder *in vivo*. The bladder was washed with saline three times, and then after 30 min incubation either (**i**) 25 μl or (**j**) 200 μl of saline containing ARL 67165 (100 μM) was slowly injected into the bladder of WT (n = 15) or VNUT-KO (n = 8) mice using PE10 tubes. The saline was collected and the ATP concentration measured. Data are mean ± SEM. Asterisks indicate a significant difference compared with the responses of VNUT-KO or control cells (*P < 0.05, **P < 0.01; Student’s t-test).

We measured extracellular ATP concentration in response to mechanical stretching. The phase-contrast images of urothelial cells and photon counting images before and after mechanical stretching are shown in Fig. 2a,b. The standard calibration curve obtained showed a high correlation between the bioluminescence intensity and ATP concentrations, with a regression analysis coefficient of 0.981 over a concentration range of 0–2.0 μM (Fig. S2). Urothelial cells in the stretch chamber were strained at an elongation of 10% (100 μm, weak) or 20% (200 μm, strong), with a stretch velocity of 100 μm/s. Stretch-evoked ATP release was dependent on the magnitude of the stretch (Fig. 2c–f). In urothelial cells obtained from VNUT-KO mice, the stretch-evoked ATP release was dramatically less than in cells from WT mice when weakly stimulated (10% elongation) (Fig. 2c, n = 6 experiments). However, the evoked ATP release in VNUT-KO cells was almost the same as that in WT cells when they were more strongly stretched (20% elongation). Using three pharmacological inhibitors, we characterized the weak and strong stretch-evoked ATP release from urothelial cells. ATP release evoked by weak stretching was significantly suppressed by the application of a vesicle-trafficking inhibitor (brefeldin A, n = 2 experiments), a vacuole H^+^-ATPase inhibitor (bafilomycin A, n = 3 experiments), or a SNARE inhibitor (botulinum toxin A, n = 3 experiments) (Fig. 2d–f). When urothelial cells were stimulated more strongly (20% elongation), ATP release was reduced by brefeldin A and bafilomycin A but not by botulinum toxin A. These results suggested that ATP release evoked by weak stretching was from vesicles (i.e. exocytosis), whereas that evoked by strong stretch was through exocytosis or other means. When urothelial cells were strongly stimulated (20% elongation), a connexin/pannexin inhibitor, carbenoxolone (100 μM), significantly inhibited ATP release from WT urothelial cells (control, 3054 ± 356 vs. 100 μM carbenoxolone, 569.2 ± 236 nM, P < 0.01) and VNUT-KO urothelial cells (control, 2712 ± 289 vs. 100 μM carbenoxolone, 270.8 ± 149 nM, P < 0.01), indicating the involvement of carbenoxolone-sensitive pathways. However, a low concentration of carbenoxolone (50 μM) inhibited ATP release from VNUT-KO urothelium (P < 0.01) but not from WT urothelium (P = 0.061) (Fig. 2g,h, n = 1 experiment). This suggests that the contribution of the connexin/pannexin pathways is increased when exocytotic release is deficient. Furthermore, we found that a pannexin-1 inhibitor, mefloquine, did not inhibit the strong, stretch-evoked ATP release (data not shown). This suggests that connexin, rather than pannexin-1, is involved in ATP release in response to strong stretching. We also measured ATP release from the mouse bladder *in vivo*. Similar to the results of the *in vitro* experiments using the culture, ATP release in VNUT-KO mouse bladders when stimulated with a smaller volume of saline (Fig. 2i, 25 μl) was significantly smaller less than that in the WT mouse bladders; however, stimulation with a higher volume of saline (Fig. 2j, 200 μl) resulted in no significant difference between the WT and VNUT-KO mice in the amount of ATP released.

### Analysis of urination behaviour using a metabolic cage

**Figure 3:**
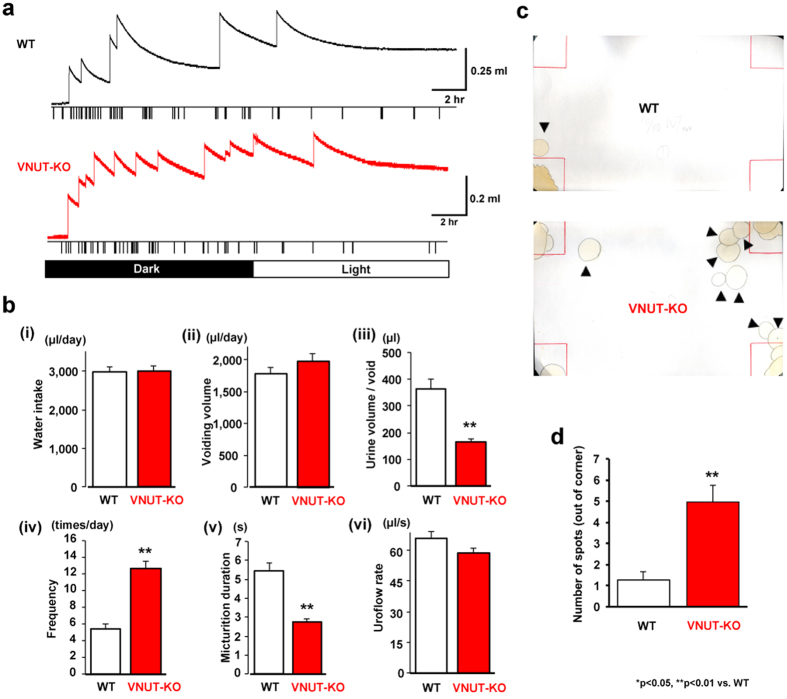
Voiding behaviour and water intake in WT and VNUT-KO mice. (**a**) Representative 24-h voided urine and water intake records from WT and VNUT-KO mice in metabolic cages. Each rapid increase in weight was evaluated as the volume of voided urine. Each spike is presented as ‘water-intake’ and indicates a decrease of 16.7 μl. (**b**) Results of 24-h frequency/volume analysis of WT (n = 13) and VNUT-KO (n = 9) mice. (i) Water intake volume per day. (ii) Urine volume per day. (iii) Mean urine volume per void. (iv) Voiding frequency per day. (v) Micturitional duration. (vi) Uroflow rate. Data are mean ± SEM. *P < 0.05, **P < 0.01 (Student’s t-test) (**c**) Spontaneous voiding pattern of unrestrained WT and VNUT-KO mice in large cages. Photographs of filter papers with urine spots from the WT or VNUT-KO mice are shown. Urine spots are distinguished by their yellowish colour and are outlined in black. Arrowheads show urine spots outside of the corner area. (**d**) Mean number of spots outside of the corner area in WT and VNUT-KO mice. Data are mean ± SEM. P < 0.01 (Student’s t-test).

Differences in bladder function between VNUT-KO and WT control mice became apparent when we analysed voluntary urination over 24 h in freely moving mice in a metabolic cage (Fig. 3a). The daily voiding frequency was significantly higher in VNUT-KO mice than in WT mice (mean ± SEM, 12.7 ± 0.85 vs. 5.42 ± 0.56 times/day, P < 0.01) (Fig. 3b iv). The mean urine volume per void was significantly lower in VNUT-KO mice than in WT mice (164 ± 12.4 vs. 366 ± 34.8 μl, P < 0.01) (Fig. 3b iii). However, the daily volumes of water intake and urine were not significantly different between the two groups (Fig. 3b i and ii). There was no significant difference in the mean urine flow rate [urine volume/duration of micturition μl/s)] between the two groups (Fig. 3b vi). Because urine volume per void was significantly lower in VNUT-KO mice (Fig. 3b iii), the duration of micturition was lower than in WT mice (2.75 ± 0.14 vs. 5.44 ± 0.40 s, P < 0.01) (Fig. 3b v). There were no differences in body, bladder, or kidney weight between the WT and VNUT-KO mice (Table S1). These results clearly show that VNUT-KO mice have a high voiding frequency but normal total daily water intake and urine volume.

### Voiding patterns in freely moving WT and VNUT-KO mice

Each mouse was placed in a large cage lined with filter paper, and its voiding behaviour was observed for 24 h. Urine spots were distinguished from the white background by their yellowish colour, and these were outlined with a pencil (Fig. 3c). The areas around the cage corners were drawn as red squares (5 × 5 cm). We counted the numbers of urine spots outside of the corner areas (Fig. 3d). There was a significantly higher number of these for VNUT-KO mice than for WT mice (P < 0.01), although there was no significant difference when the animals were placed in smaller cages (WT, n = 5; VNUT-KO, n = 5; P = 0.13). We also tested locomotor activity in an open-field area. We found no significant differences in the total distance travelled or velocity between WT and VNUT-KO mice (data not shown).

### Urodynamic analysis using cystometrograms

**Figure 4:**
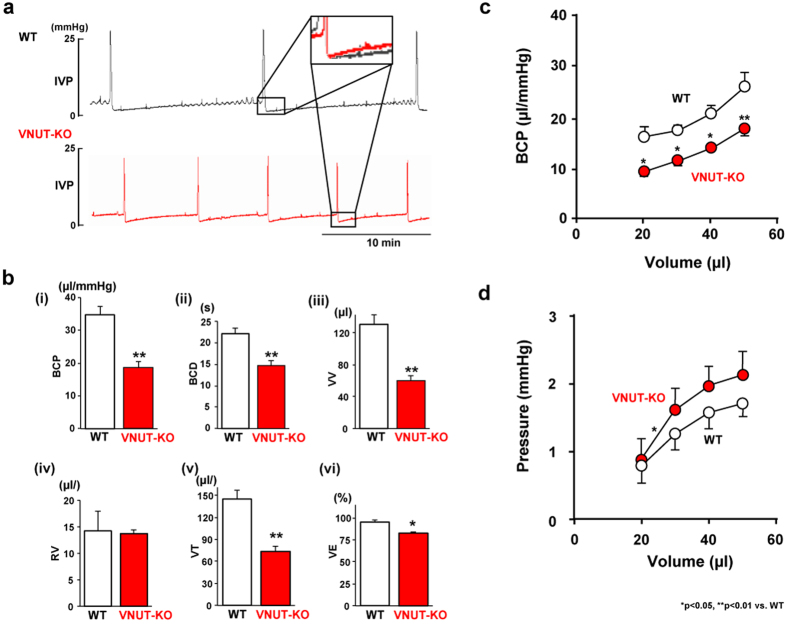
Cystometry in decerebrate, unanaesthetised WT and VNUT-KO mice. (**a**) Cystometry chart of decerebrate, unanaesthetised mice. Typical bladder activity during continuous saline infusion is shown by CMGs (infusion rate: 10 μl/min) from a WT and a VNUT-KO mouse. The inset shows magnified superimposed images from the two CMGs to show the difference in intravesical pressure during the early phase of bladder filling. (**b**) Results of cystometry in decerebrate, unanaesthetised WT (n = 7) and VNUT-KO (n = 7) mice. (i) Bladder compliance (BCP), (ii) bladder contraction duration (BCD), (iii) voided volume (VV), (iv) residual volume (RV), (v) volume threshold (VT) and (vi) voiding efficacy (VE). BCD (s) is the duration from the pressure threshold (PT, mmHg), which is the intraluminal pressure required to induce a voiding contraction, to resting pressure (RP, mmHg), which is the lowest pressure immediately after a voiding contraction^54^. VV (μl) is the volume fluid voided from the urethral meatus^60^. RV (in μl) is the volume of fluid remaining in the intravesical space. VT (μl) is the volume that induces micturition, calculated as the sum of the voided and residual volume. From these values, voiding efficiency (VE; %) was estimated as follows: VE = VV/VT × 100. The other parameters evaluated were pressure at volume threshold for inducing micturition contraction (mmHg), maximal voiding pressure (mmHg), closing peak pressure (mmHg), and bladder compliance (μl/mmHg)^60^,^61^. Bladder compliance (BCP) was calculated as the ratio of the infused volume to the pressure difference between the post-void resting pressure and the following pressure at the volume threshold for inducing micturition contraction. (**c**) Intravesical volume: mean BCP analysis (from 20 to 50 μl). (**d**) Intravesical volume: pressure relation analysis (from 20 to 50 μl). Data are mean ± SEM. Asterisks show significant differences in response between VNUT-KO and WT mice (*P < 0.05, **P < 0.01) (Mann–Whitney U test). An asterisk in (**d**) indicates a significant difference in the slope (from 20 to 30 μl) between the groups (*P < 0.05).

To evaluate the role of VNUT in reflex urine voiding, cystometrograms (CMGs) were performed during slow, continuous bladder filling in decerebrate, unanaesthetised animals (Fig. 4a). The intercontraction intervals of VNUT-KO mice were lower than those of WT mice during continuous saline infusion (infusion rate: 10 μl/min) (Fig. 4a). VNUT-KO mice had lower BCP than WT mice (P < 0.01, Fig. 4b (i), Table 1). Significant differences in BCP were observed at volumes between 20 and 50 μl (Fig. 4c). Voided volume (P < 0.01) and volume threshold (P < 0.01) in VNUT-KO mice were significantly lower than in WT mice (Fig. 4b (iii), (v) and Table 1). VNUT-KO mice had a shorter duration of bladder contraction than WT mice (P < 0.01, Fig. 4b (ii) and Table 1). Voiding efficacy in VNUT-KO mice was significantly lower than in WT mice (P < 0.01, Fig. 4b (vi) and Table 1). A low voiding efficacy value may be because of a small bladder volume at the start of voiding. Residual urine volume, maximal voiding pressure, pressure threshold and resting pressure in VNUT-KO mice were comparable to those in WT mice (Fig. 4b (iv) and Table 1). No difference in intravesical pressure was detected between VNUT-KO and WT mice over a range of intravesical volumes (Fig. 4d). However, interestingly, there was a significant difference in the slope of pressure elevation relative to an intravesical volume increase from 20 to 30 μl (P = 0.041) between the VNUT-KO and WT mice. However, the slope of pressure increase was the same in the two groups when intravesical volume increased from 30 to 40 μl (P = 0.810) or from 40 to 50 μl (P = 0.842) (Fig. 4d). These results clearly showed that the increased urinary frequency in VNUT-KO mice was associated with phenotypes with low BCP during the early stage of bladder filling.

### Anatomic and functional analysis of urothelial membrane trafficking

**Figure 5:**
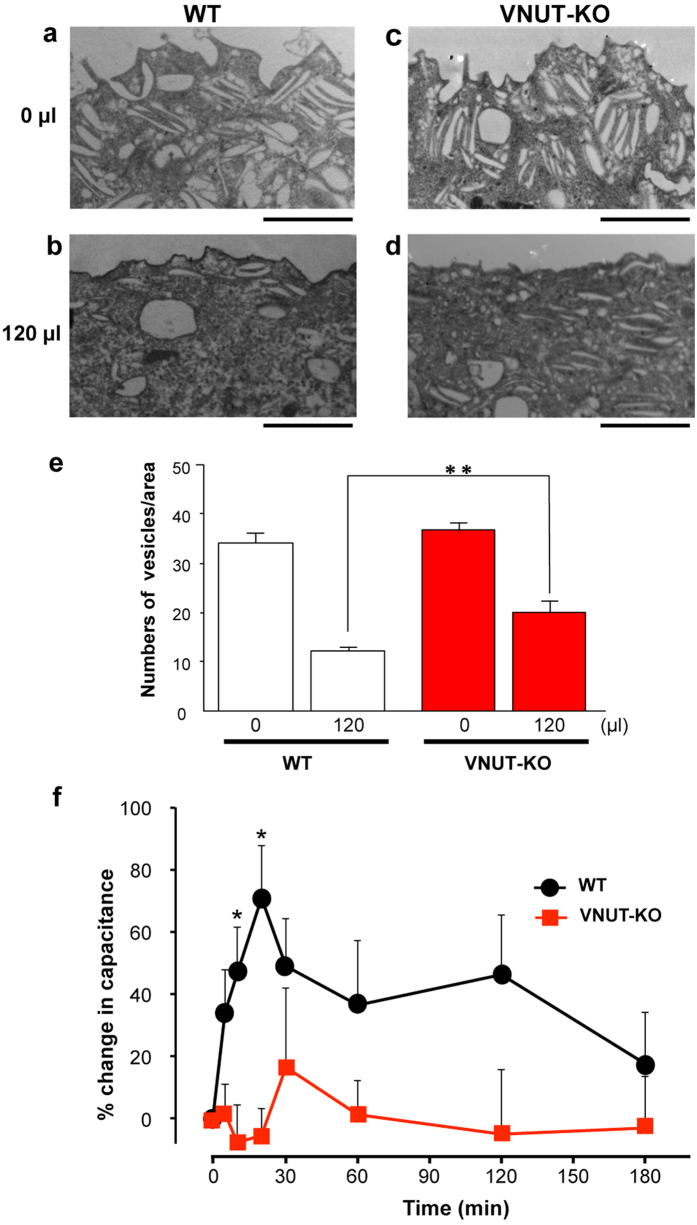
Anatomic and functional analysis of urothelial membrane trafficking. (**a–e**) Transmission electron microscopic analysis of the umbrella cells apical poles in the WT and VNUT-KO mouse bladder. WT (**a,b**) and VNUT-KO (**c,d**) mice were catheterized, and the urine contained in the bladder was drained. After incubation for 30 min, control bladders (**a,c**), for which no saline was added, and the saline-filled bladders (**b,d**), which were filled with 120 μl of normal saline at a rate of 1.6 μl/min, were processed and imaged. Scale bar, 2.0 μm. (**e**) The number of FDVs in randomly placed 2 μm radius circles in the apical cell layer. Data are the mean ± SEM. **P < 0.01 (Student’s t-test). (**f**) The time-course of changes in membrane capacitance with hydrostatic pressure measured by an Ussing chamber. Isolated mouse bladder tissue was exposed to pressure across the mucosal side at t = 0 and the capacitance was recorded. Values show mean changes in capacitance ± SEM (WT, n = 7; VNUT-KO, n = 4). Asterisks show significant differences in capacitance between WT and VNUT-KO bladders (*P < 0.05).

Previous studies have shown that increased hydrostatic pressure or slow filling of intact bladders causes ATP release. This release is followed by the activation of P2 X 2 and P2 X 3 receptors expressed by apical urothelial cells and the fusion and/or exocytosis of discoidal/fusiform vesicles located in the cytoplasm of umbrella cells. This change can result in an increase in the apical urothelial area^12^. Consistent with these observations, we found that without any saline infusion, there was an abundance of discoidal/fusiform vesicles in the umbrella cells of both WT and VNUT-KO mice (Fig. 5a,c). When the mouse bladders were slowly filled with saline (1.6 μl/min for 75 min), the number of discoidal/fusiform vesicles in the apical cytoplasm of WT mice was decreased (Fig. 5b). However, under the same conditions, the number of vesicles in VNUT-KO mice was significantly different compared with that in WT mice (Fig. 5d,e). This 120 μl infusion volume was based on the average micturition volume per unit of time for VNUT-KO mice [Fig. 3b (iii)]. These results indicated that the urothelium releases ATP mainly through exocytosis, causing the fusion/exocytosis of discoidal/fusiform vesicles and incremental changes in the apical surface of the bladder at the filling phase until a bladder volume of 120 μl was reached.

We also performed functional analyses of urothelial membrane trafficking of the bladder using the Ussing chamber. Figure 5f shows the time-course of changes in membrane capacitance with hydrostatic pressure. The increase in the membrane capacitance was significantly lower in the VNUT-KO bladder than in the WT bladder.

## Discussion

Our study showed that VNUT was highly expressed in all layers of the bladder urothelium and in primary urothelial cell cultures of WT mice. This expression was eliminated in VNUT-KO mice, which exhibited increased voiding frequency and abnormal patterns of voluntary voiding as well as decreased intercontraction intervals and BCP in decerebrate animals during cystometry. However, the amplitude of reflex bladder contractions and post-micturition residual volume were the same in VNUT-KO mice as they were in WT mice. In VNUT-KO mice, ATP release from urothelial cell cultures evoked by weak stretching (10% elongation) was significantly reduced compared with WT mice; however, ATP release in response to stronger stretching (20% elongation) was not affected. These results suggest that ATP is released from urothelial cells by multiple mechanisms activated at different levels of bladder distension. They also suggest that VNUT-mediated urothelial release of ATP plays an important role in urine storage mechanisms that promote relaxation of the bladder during the early stages of filling.

Previous studies by other investigators have shown that ATP release from the urothelium is dependent in part on a vesicular, exocytotic mechanism that is suppressed by botulinum toxin^13^,^27^,^28^. Consistent with these data, we found that the VNUT-mediated release of ATP that occurred under low stretch conditions was sensitive to botulinum neurotoxin A and a vesicle-trafficking inhibitor (brefeldin A) or a vacuole H^+^-ATPase inhibitor (bafilomycin A). Fluorescence microscopy showed that the fluorescent ATP analogue MANT-ATP accumulated in VNUT-RFP-positive vesicles (Fig. 1l). Quinacrine, which stains acidic vesicles, was also detected in VNUT-RFP-positive vesicles in urothelial cells. The mechanical stimulation of these cells reduced quinacrine staining (Fig. S1), consistent with a putative vesicular release mechanism.

When urothelial cells were stimulated by strong stretching (20% elongation), ATP release was reduced by brefeldin A and bafilomycin A, but not by botulinum neurotoxin A. This suggests that ATP release evoked by strong stimulation occurs by other means, such as through connexin or pannexin channels^12^,^17^, in addition to exocytosis. Mefloquine, a pannexin-1 inhibitor, did not inhibit the strong stretch-evoked ATP release. However, carbenoxolone (100 μM), a connexin/pannexin inhibitor, significantly inhibited ATP release induced by this stimulation in WT and VNUT-KO urothelial cells. This finding indicates that there were non-exocytotic ATP releasing pathways, for example connexin-mediated pathway, besides exocytosis in the strong stretching condition. A low concentration of carbenoxolone (50 μM) suppressed ATP release from VNUT-KO but not WT urothelium (Fig. 2g,h). This suggests that the sensitivity to blocking effect of carbenoxolone or the contribution of the connexin pathway to ATP release increases when the exocytotic machinery is deficient.

Exocytosis is the most sophisticated mechanism for secretion of chemical transmitters. It is generally observed in professional secretory cells, such as neurons, as well as endocrine and exocrine cells. However, exocytosis also occurs in other types of non-neural cells, such as osteoblasts^29^, glial cells^30^,^31^,^32^ and several types of epithelial cells^33^, including urothelial cells of the ureter^23^. Multiple release mechanisms, as proposed in the present study, for bladder urothelial cells have also been detected in other cells^34^. However, the activation of different release mechanisms by different stimuli in bladder urothelial cells is a novel finding of our study.

Multiple observations have focused attention on the potential excitatory function of ATP released from the urothelium^35^. Bladder afferent nerves that are located in proximity to the urothelium express excitatory purinergic receptors (P2 X2, P2 X3). Exogenous ATP excites these receptors and induces bladder overactivity. The knockout of the P2X receptors induces bladder hypoactivity, while drugs that block P2X receptors suppress certain types of bladder overactivity^10^,^11^. Therefore, the finding that VNUT-KO mice, which showed a decreased release of urothelial ATP, had a reduced BCP and increased voiding frequency instead of bladder hypoactivity was unexpected. This finding suggests that ATP released from the urothelium participates in inhibitory as well as excitatory mechanisms that control bladder function. Urothelial cells express multiple types of purinergic receptors, which can be activated by an autocrine-based mechanism in an autocrine manner by ATP released in the urothelium^35^. The activation of urothelial P2X receptors can trigger vesicle trafficking in urothelial cells^12^ and further enhance the release of ATP^36^. This autocrine mechanism could also enhance the release of other chemicals that may play a role in urothelial signalling in the bladder.

One substance that may be involved in the putative VNUT-activated purinergic inhibitory mechanism mentioned above is urothelial-derived inhibitory factor^37^,^38^,^39^, which can be released by the activation of urothelial muscarinic^38^ or purinergic receptors^40^ and acts on bladder smooth muscle to suppress muscle contractions. Urothelial-derived inhibitory factor has not been identified; however, it is known to be one of the many common signalling molecules affecting the bladder muscle.

**Figure 6:**
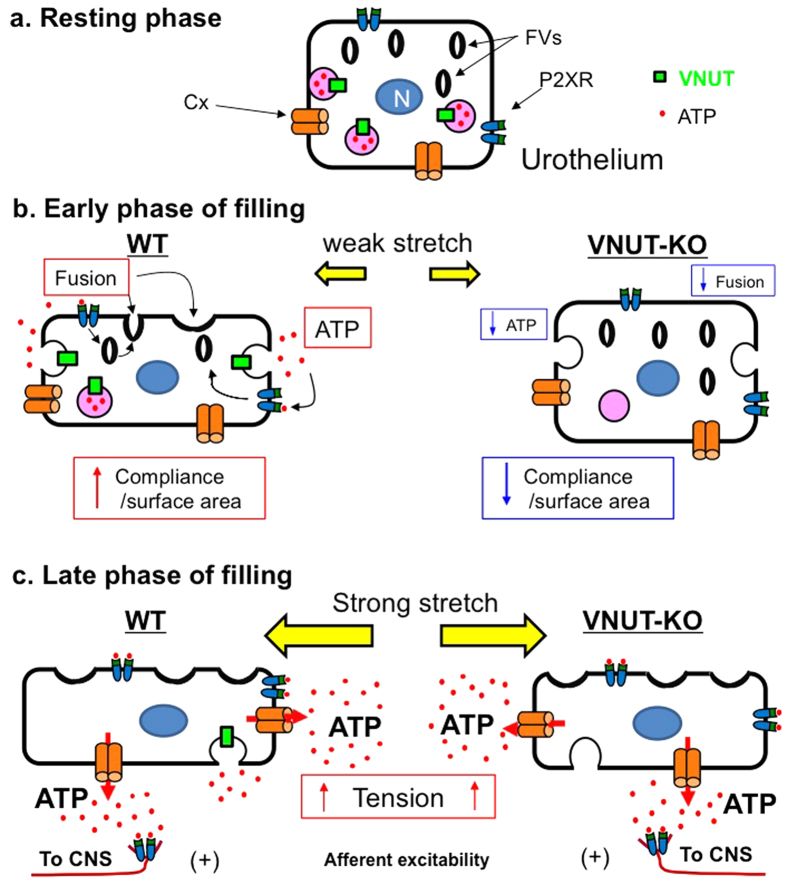
A model showing bladder function mediated by urothelial release of ATP in each bladder-filling phase. (**a**) Resting phase of bladder urothelium. ATP accumulates in vesicles with VNUT. Fusiform vesicles are abundant in the cytoplasm. Connexin (Cx) and P2X receptors (P2XR) are located on the urothelial cell. For simplicity, multiple layers of urothelium, bladder smooth muscle, suburothelial interstitial cells or myofibroblasts, other receptors and transmitters including urothelium-derived inhibitory factor have been omitted. (**b**) During the early phase of bladder filling, small increases in intravesical pressure and bladder distension stimulate a VNUT-mediated release of ATP from the urothelium. This ATP acts via an autocrine mechanism that induces fusion of fusiform/discoidal vesicles (FDVs) with the urothelial plasma membrane to increase the surface area. These mechanisms lead to increase in bladder compliance and lower intravesical pressure during filling. (**c**) The late filling phase produces greater increases in intravesical pressure and increased FDVs fusion with the plasma membrane. This is accompanied by a reduction in cytoplasmic FDVs numbers, stimulating further ATP release from the urothelium. In this phase, ATP release may occur through multiple mechanisms involving Cx hemichannels and vesicular release. The greater amount of ATP released stimulates P2 receptors on interstitial cells or myofibroblasts or afferent nerves. This process increases nerve firing and the information transmission about bladder filling to the central nervous system, where bladder filling sensations are triggered. In VNUT-KO mice, elimination of VNUT-mediated ATP release during the early phase of bladder filling reduces putative mechanisms that increase bladder compliance. This process then reduces compliance and leads to increase in muscle and/or mucosal tension, which in turn leads to increased afferent nerve firing and bladder sensations at a lower volume.

The effect of VNUT on BCP may involve multiple mechanisms. During the early phase of bladder filling (Fig. 6b), VNUT-mediated ATP release acts on urothelial P2X receptors to induce fusiform vesicle fusion with the plasma membrane, leading to membrane surface expansion^12^ (Fig. 5) and a reduction in epithelial membrane tension^41^. Another possibility is that VNUT-mediated ATP release could act via an autocrine mechanism to release inhibitory factor, which in turn regulates smooth muscle directly or indirectly via suburothelial interstitial cells or myofibroblasts. This mechanism is consistent with the results of a previous study, which showed that ATP releases inhibitory factor in the rat urinary bladder^40^. A third possibility is that adenosine, a metabolite of ATP, stimulates P1 purinoceptors located in bladder smooth muscle^42^ and induces relaxation^43^,^44^. This mechanism of purinergic bladder relaxation has been proposed for ATP release from nerves and may also occur following VNUT-mediated release of ATP from the urothelium. The elimination of any or all of these putative mechanisms may induce an increase in muscle and/or mucosal tension, leading to increased afferent excitability at a lower volume, followed by an increased voiding frequency (Fig. 6).

An effect of VNUT in indirectly regulating voiding frequency by modulating BCP is consistent with the effects of mirabegron, a β3 adrenergic receptor agonist used to treat an overactive bladder. This drug increases BCP and the intervoid interval without affecting the voiding function^45^. Therefore, by directly activating β3 adrenergic receptors in the bladder smooth muscle, mirabegron mimics the proposed VNUT-mediated effect on BCP and the voiding interval. These findings suggest that VNUT-mediated ATP regulates detrusor and intravesical pressure during the storage phase by an action on BCP similar to that of sympathetic nerve activity. Mirabegron may also promote urine storage by activating urothelial β3 adrenergic receptors to induce the release of inhibitory factor^46^ or by directly inhibiting afferent nerve activity^47^.

During the late phase of bladder filling (Fig. 6c), we propose that substantial ATP released from the urothelium via multiple mechanisms acts on several different P2 receptors (P2XR and P2YR) in afferent nerves and/or on interstitial cells or myofibroblasts. This elicits a further increase in afferent signalling to the central nervous system that in turn induces a micturition reflex.

VNUT-mediated ATP release may be less important during this phase of the voiding cycle compared with its role in the early filling phase because the intravesical pressure required to induce voiding was unchanged in VNUT-KO mice. However, VNUT-mediated ATP release could also affect bladder sensation during the filling phase based on the increased number of urine spots outside of the corner areas in VNUT-KO mice following a change in cage size, which could not be explained by only a low BCP. The other reason was that VNUT expression levels in bladder mucosal biopsies from patients with lower urinary tract symptom (LUTS) negatively correlated with bladder volume at First desire to void, one of the sensations experienced during the filling phase^48^.

There was no significant change in VNUT-KO mice for various parameters of voiding, including maximal voiding pressure and residual urine volume. Therefore, there is a possibility that VNUT-KO mice may have a lack of warning sensation^49^ in addition to low BCP and suddenly feeling a strong sensation. This strong sensation may be triggered by ATP released from pathways other than exocytosis, causing urination before returning to the corner areas.

In the present study, we used conventional VNUT-KO mice, and thus cannot exclude the possibility that the deficiency of non-bladder or non-urothelial VNUT might affect the ATP-mediated bladder functions seen in the study. However, based on the present study’s various *in vivo*, *ex vivo*, and *in vitro* experiments, we tentatively hypothesized that VNUT-deficiency in the urothelium affected the urine storage mechanisms in the bladder in the early stages of filling, thereby leading to a reduction in bladder compliance. Verification of this hypothesis must await further studies using urothelium-specific conditional VNUT-KO mice.

In conclusion, VNUT-mediated ATP release from urothelial cells appears to play an inhibitory role to promote BCP and urine storage in normal mice. ATP release via VNUT in patients with LUTS may potentially play an excitatory role in sensory mechanisms that increase the sensation of bladder filling at a low bladder volume, thereby inducing increased urinary frequency and nocturia. Further studies are required to examine the effect of pathology on the functions of VNUT in the bladder.

## Materials and Methods

### Animals

All animals in this study were obtained, housed, cared for and used in accordance with the “Guiding Principles in the Care and Use of Animals in the Field of Physiologic Sciences” published by the Physiologic Society of Japan. In addition, all experimental protocols were approved by the Animal Care Committee of the University of Yamanashi (Chuo, Yamanashi, Japan). C57BL/6 mice (SLC, Shizuoka, Japan) and VNUT-KO mice backcrossed (for eight generations) on a C57BL/6 background were used. VNUT-KO mice were generated by homologous recombination in mouse embryonic stem cells containing a genetic deletion of the first four transmembrane domains of VNUT, as previously reported^50^. The VNUT-KO mice appeared to be normal when compared to WT mice in terms of weight gain, body size, morphology, food intake, water intake, oxygen consumption, carbon dioxide emission, respiratory exchange ratio, locomotor activity, and open-field and plus-maze behaviors. For genotyping using polymerase chain reaction (PCR), the following primers were used: WT forward, 5′CTA TGT GTA GCC CTG GAT GG-3′; WT reverse, 5′GTG TAC CCT TCG GGG AAA GT-3′; VNUT-KO forward (corresponding to a partial sequence inside of the PGK promoter that drives the Neo gene), 5′CTT GTG TTT GGG ATC CTG GT-3′ and VNUT-KO reverse, 5′GGG AGG ATT GGG AAG ACA AT-3′. Mice were housed in a specific pathogen-free environment with a 12-h light and 12-h dark cycle and allowed water and standard food freely. All experiments were performed using 8- to 12-week-old male mice.

### Human subjects

Bladder specimens were harvested from 15 male patients with prostate cancer or benign prostatic hyperplasia who had undergone surgery at a single hospital (Kawahara Nephro-Urology Clinic, Kagoshima, Japan). The present study was approved by the Institutional Review Board of the Kawahara Nephro-Urology Clinic (# 62) and was conducted according to the principles expressed in the Declaration of Helsinki. All specimens were used only after each patient had provided fully informed consent.

### Drugs and treatments

The compound 2′/3′-(N-methylanthraniloyl)-adenosine-5′-triphosphate, triethylammonium salt (MANT-ATP; Jena Bioscience, Jena, Germany), quinacrine (Research Organics, Cleveland, OH), carbenoxolone (Sigma-Aldrich Japan), brefeldin A (Wako, Japan), bafilomycin A1 (Sigma-Aldrich Japan) and botulinum toxin type A (Botox®; Allergan, Irvine, CA) were used.

### Primary urothelial cell cultures

Urothelial cultures were prepared as previously described^15^.

### Immunostaining

Whole bladders were excised from mice and primary urothelial cell cultures were fluorescently immunostained with the primary antibody rabbit anti-VNUT (a self-made antibody)^24^ or anti-CK7 (Dako Denmark A/S, Glostrup, Denmark) as previously described^15^. For morphological analysis, bladders were weighed, fixed in formalin, embedded in paraffin and cut into 5-μm sections, which were stained with H&E.

### Preparation of the membrane fraction at the bladder

Bladders were isolated from WT and VNUT-KO mice. After washing with 10 mM MOPS-Tris (pH 7.0) containing 300 mM sucrose, bladders were homogenized in 10 mM MOPS-Tris (pH 7.0) containing 300 mM sucrose, 5 mM EDTA, 10 mg/ml pepstatin A and 10 mg/ml leupeptin. The homogenate was centrifuged (12,000 g, 4 °C, for 10 min) to remove organelles and cellular debris. The resultant supernatant was collected and centrifuged again (150,000 g, 4 °C, for 1 h). The pellet (membrane fraction) was suspended with the same buffer.

### Visualization of ATP vesicles

Urothelial cells were transfected with VNUT-RFP plasmid (50 ng/ml) using Lipofectamine RNAi MAX (Life Technologies Corp., Tokyo, Japan) for 48 h. The fluorescent ATP analogue MANT-ATP (200 μM for 12 h) was then applied for co-localization, and quinacrine (0.75 μM for 15 min) was used for kinetic analysis of the ATP vesicles. A single urothelial cell was stimulated with a glass micropipette using a micromanipulator (Narishige, Tokyo, Japan).

### ATP measurement

Mechanical stretching experiments and photon imaging of ATP release were performed as previously described^15^,^51^. In brief, an elastic silicone stretch chamber (STB-CH-04, STREX, Osaka, Japan) and an extension device (STB150, STREX) were set on a photon imaging system, and the chamber medium was replaced with extracellular solution containing a luciferase reagent (ATP bioluminescence assay kit CLS II, Roche Diagnostics, Basel, Switzerland). ATP bioluminescence during stretch stimulation was detected and visualized with a VIM camera (C2400-35, Hamamatsu Photonics, Hamamatsu, Japan). The standard calibration curve yielded a correlation coefficient for bioluminescence vs. ATP concentration of 0.981 over a concentration range of 0 nM to 2 μM (Fig. S2). To measure ATP release measurement *in vivo*, 25 μl or 200 μl of saline containing ARL 67165 (100 μM) was slowly injected into the ureters ligated-bladder of WT or VNUT-KO mice using a PE-10 tube (Clay-Adams, Parsippany, NJ). The saline was collected and the ATP concentration measured by using the luciferase reagent. Although the previous report by Yu^52^ showed that extracellular ATP concentration and its distribution in the urothelium was affected by ectonucleotidases, the activity of these enzymes was unaffected by the deletion of VNUT (data not shown).

### Metabolic cages and voluntary voiding pattern

VNUT-KO and age-matched WT mice acclimatized for two days were placed in a newly designed, soundproof metabolic cage with a special mesh (patent license no.: 2009–187420, Mitsubishi Kumamoto, Japan), which passed urine but caught faeces and/or food; a drink sensor (DS-1; Shin Factory, Fukuoka, Japan) and a digital scale (GX-400; A&D Co., Tokyo, Japan)^53^. Voiding frequency, volume per void and water intake volume were recorded for 72 h. Individual mice were placed in a 39 × 24 cm (large) or 26 × 19 cm (small) cage. The urine output was collected for 24 h in the entire area of the cage on covered filter paper (Bio-Rad, Hercules, CA). These areas were photographed and the number of urine spots outside of the corner areas was counted.

### Urodynamic analysis using CMGs

Pre-collicular decerebration was performed according to a previously published method^54^.

### Bladder filling and electron microscopy

A PE-10 tube was gently guided through small incision in the urethra into the bladder lumen, and then the incision was closed with a 7-0 nylon ligature. Following bladder drainage, the bladder was incubated for 30 min and then either excised and processed for transmission electron microscopy (TEM) or slowly filled with a normal saline at a rate of 1.6 μl/min for 75 min. For TEM (H-7500; Hitachi, Tokyo, Japan), tissue fixation, embedding, sectioning and electron microscopy were performed as previously reported^55^. To quantify the number of FDVs, the number of FDVs was counted in three randomly selected 2.0 μm radius per image, placed just below the surface of the apical cell.

### Functional analysis of membrane trafficking using an Ussing chamber

Urinary bladders were harvested from euthanized mice through an abdominal incision and then cut open. The tissue was maintained in Krebs solution containing the following (in mM): NaCl 110, NaHCO_3_ 25, KCl 5.8, MgSO4 1.2, KH_2_PO_4_ 1.2, glucose 11, and CaCl_2_ 2. The solution was buffered at pH 7.4 by gassing with a mixture of 95% O_2_/5% CO_2_. Each bladder was mounted onto a modified Ussing chamber P2310 tissue slider (Physiologic Instruments, San Diego, CA) with a 0.5 cm diameter opening surrounded by eight 3.5-mm sharp pins placed at approximately 3 mm from the opening. The tissue sliders were assembled into the chambers of an EM-CSYS Ussing system (Physiologic Instruments) equipped with a heat block for temperature control. The mucosal and serosal hemichambers were each filled with 3 ml of Krebs solution. The temperature inside the chambers was kept at 37 °C. The hemichambers were continuously bubbled with 95% O_2_/5% CO_2_. The tissue was allowed to equilibrate for at least 45 min before the experimental manipulations. Capacitance was measured as a means of estimating surface area (where 1 μF is equivalent to 1 cm^2^ actual membrane area) as described previously^56^,^57^. To measure capacitance, a square current pulse of 1 μA, generated from within the pClamp software (Ver. 10.0 Molecular Devices) and passed from the Digidata 1550 A/D converter (Molecular Devices) to a VCC MC6 current/voltage clamp (Physiologic Instruments, San Diego, CA, USA), was applied across the tissue for 200 ms with a delay of 25 ms. The voltage response of the tissue was digitized by the Digidata 1550 A/D converter and recorded every 60 s using the pClamp software. The time constant, τ, was obtained by fitting the voltage response to a single exponential function by using the pClamp software. The capacitance was determined using the formula C = τ/R, where C is the capacitance and R is the resistance. The resistance was determined by dividing the amplitude of the steady-state voltage response by the amplitude of the square current pulse^12^,^58^,^59^. The volume in the mucosal hemichamber was increased by sequentially adding Krebs solution (at 4.0 ml/h up to 2.0 ml), which augmented the hydrostatic pressure.

### Statistical analysis

Experimental results are expressed as mean ± SEM. The statistical significance of differences between two groups was determined by the Student’s t-test or Mann–Whitney U test. A P value < 0.05 was considered significant.

## Additional Information

**How to cite this article**: Nakagomi, H. *et al*. Urothelial ATP exocytosis: regulation of bladder compliance in the urine storage phase. *Sci. Rep*. **6**, 29761; doi: 10.1038/srep29761 (2016).

## Supplementary Material

Supplementary Information

## Figures and Tables

**Table 1:** Comparisons between WT and VNUT-KO mice in cystometric parameters.

	**PT**	**MVP**	**RP**	**BCP**	**BCD**	**VV**	**RV**	**VT**
	**(mmHg)**	**(mmHg)**	**(mmHg)**	**(μL/mmHg)**	**(s)**	**(μL)**	**(μL)**	**(μL)**
WT	4.00 ± 0.391	23.3 ± 2.5	−0.3 ± 0.13	34.8 ± 2.64	22.1 ± 1.32	131 ± 12.1	14.3 ± 3.72	145 ± 11.6
	(2.47–5.03)	(13.6–32.8)	(−0.90–0.13)	(24.8–44.5)	(16.9–26.0)	(72–174)	(7–36)	(83–181)
VNUT-KO	3.32 ± 0.25	20.0 ± 1.27	−0.15 ± 0.23	18.6 ± 1.77**	14.7 ± 1.17**	60 ± 6.75**	13.7 ± 0.68	73.7 ± 6.64
	(2.44–4.11)	(16.9–26.3)	(−0.86–1.07)	(13.0–26.3)	(11.5–19.1)	(94–39)	(11–16)	(53–106)

Note. PT, pressure threshold for inducing micturition contraction; MVP, maximal voiding pressure; RP, resting pressure; BCP, bladder compliance. BCD, bladder contraction duration; VV, voided volume; RV, residual volume; VT, volume threshold. Numbers in parentheses are ranges. Statistical difference from WT: *P < 0.05 and **P < 0.01 (Student’s t-test). Values are expressed as means ± SEM (n = 7).

## References

[b1] Chapple C. Overview on the lower Urinary Tract. Handb Exp Pharmacol 202, 1–14 (Springer, 2011). 21290219 10.1007/978-3-642-16499-6_1

[b2] Abrams P. . The standardisation of terminology in lower urinary tract function: report from the standardisation sub-committee of the International Continence Society. Urology 61, 37–49 (2003). 12559262 10.1016/s0090-4295(02)02243-4

[b3] Yokoyama O. . Bladder compliance in patients with benign prostatic hyperplasia. Neurourol Urodyn 16, 19–27; discussion 28–19 (1997). 9021787 10.1002/(sici)1520-6777(1997)16:1<19::aid-nau2>3.0.co;2-h

[b4] Apodaca G. The uroepithelium: not just a passive barrier. Traffic 5, 117–128 (2004). 15086788 10.1046/j.1600-0854.2003.00156.x

[b5] Birder L. A. & de Groat W. C. Mechanisms of disease: involvement of the urothelium in bladder dysfunction. Nat Clin Pract Urol 4, 46–54 (2007). 17211425 10.1038/ncpuro0672 PMC3119256

[b6] Everaerts W., Gevaert T., Nilius B. & De Ridder D. On the origin of bladder sensing: Tr(i)ps in urology. Neurourol Urodyn 27, 264–273 (2008). 17849480 10.1002/nau.20511

[b7] Araki I. . Roles of mechanosensitive ion channels in bladder sensory transduction and overactive bladder. Int J Urol 15, 681–687 (2008). 18462357 10.1111/j.1442-2042.2008.02052.x

[b8] Birder L. A. More than just a barrier: urothelium as a drug target for urinary bladder pain. Am J Physiol Renal Physiol 289, F489–495 (2005). 16093424 10.1152/ajprenal.00467.2004

[b9] de Groat W. C. The urothelium in overactive bladder: passive bystander or active participant? Urology 64, 7–11 (2004). 15621221 10.1016/j.urology.2004.08.063

[b10] Cockayne D. A. . Urinary bladder hyporeflexia and reduced pain-related behaviour in P2X3-deficient mice. Nature 407, 1011–1015 (2000). 11069181 10.1038/35039519

[b11] Vlaskovska M. . P2X3 knock-out mice reveal a major sensory role for urothelially released ATP. J Neurosci 21, 5670–5677 (2001). 11466438 10.1523/JNEUROSCI.21-15-05670.2001 PMC6762653

[b12] Wang E. C. . ATP and purinergic receptor-dependent membrane traffic in bladder umbrella cells. J Clin Invest 115, 2412–2422 (2005). 16110327 10.1172/JCI24086 PMC1187935

[b13] Hanna-Mitchell A. T. . Non-neuronal acetylcholine and urinary bladder urothelium. Life Sci 80, 2298–2302 (2007). 17363007 10.1016/j.lfs.2007.02.010 PMC3085916

[b14] Kruse R., Save S. & Persson K. Adenosine triphosphate induced P2Y2 receptor activation induces proinflammatory cytokine release in uroepithelial cells. J Urol 188, 2419–2425 (2012). 23088987 10.1016/j.juro.2012.07.095

[b15] Mochizuki T. . The TRPV4 cation channel mediates stretch-evoked Ca^2+^ influx and ATP release in primary urothelial cell cultures. J Biol Chem 284, 21257–21264 (2009). 19531473 10.1074/jbc.M109.020206 PMC2755849

[b16] Stout C. E., Costantin J. L., Naus C. C. & Charles A. C. Intercellular calcium signaling in astrocytes via ATP release through connexin hemichannels. J Biol Chem 277, 10482–10488 (2002). 11790776 10.1074/jbc.M109902200

[b17] Timoteo M. A. . ATP released via pannexin-1 hemichannels mediates bladder overactivity triggered by urothelial P2Y6 receptors. Biochem Pharmacol 87, 371–379 (2014). 24269631 10.1016/j.bcp.2013.11.007

[b18] Anderson C. M., Bergher J. P. & Swanson R. A. ATP-induced ATP release from astrocytes. J Neurochem 88, 246–256 (2004). 14675168 10.1111/j.1471-4159.2004.02204.x

[b19] Darby M., Kuzmiski J. B., Panenka W., Feighan D. & MacVicar B. A. ATP released from astrocytes during swelling activates chloride channels. J Neurophysiol 89, 1870–1877 (2003). 12686569 10.1152/jn.00510.2002

[b20] Pellegatti P., Falzoni S., Pinton P., Rizzuto R. & Di Virgilio F. A novel recombinant plasma membrane-targeted luciferase reveals a new pathway for ATP secretion. Mol Biol Cell 16, 3659–3665 (2005). 15944221 10.1091/mbc.E05-03-0222 PMC1182305

[b21] Ohshima Y. . gamma-Irradiation induces P2X(7) receptor-dependent ATP release from B16 melanoma cells. Biochim Biophys Acta 1800, 40–46 (2010). 19854240 10.1016/j.bbagen.2009.10.008

[b22] Reisin I. L. . The cystic fibrosis transmembrane conductance regulator is a dual ATP and chloride channel. J Biol Chem 269, 20584–20591 (1994). 7519611

[b23] Knight G. E., Bodin P., De Groat W. C. & Burnstock G. ATP is released from guinea pig ureter epithelium on distension. Am J Physiol Renal Physiol 282, F281–288 (2002). 11788442 10.1152/ajprenal.00293.2000

[b24] Sawada K. . Identification of a vesicular nucleotide transporter. Proc Natl Acad Sci USA 105, 5683–5686 (2008). 18375752 10.1073/pnas.0800141105 PMC2311367

[b25] Mihara H., Boudaka A., Sugiyama T., Moriyama Y. & Tominaga M. Transient receptor potential vanilloid 4 (TRPV4)-dependent calcium influx and ATP release in mouse oesophageal keratinocytes. J Physiol 589, 3471–3482 (2011). 21540339 10.1113/jphysiol.2011.207829 PMC3167111

[b26] Sesma J. I. . Vesicular nucleotide transporter regulates the nucleotide content in airway epithelial mucin granules. Am J Physiol Cell Physiol 304, C976–984 (2013). 23467297 10.1152/ajpcell.00371.2012 PMC3651637

[b27] Smith C. P., Vemulakonda V. M., Kiss S., Boone T. B. & Somogyi G. T. Enhanced ATP release from rat bladder urothelium during chronic bladder inflammation: effect of botulinum toxin A. Neurochem Int 47, 291–297 (2005). 15970360 10.1016/j.neuint.2005.04.021

[b28] Collins V. M. . OnabotulinumtoxinA significantly attenuates bladder afferent nerve firing and inhibits ATP release from the urothelium. BJU Int 112, 1018–1026 (2013). 23937318 10.1111/bju.12266

[b29] Romanello M. . Autocrine/paracrine stimulation of purinergic receptors in osteoblasts: contribution of vesicular ATP release. Biochem Biophys Res Commun 331, 1429–1438 (2005). 15883034 10.1016/j.bbrc.2005.03.246

[b30] Koizumi S., Fujishita K., Tsuda M., Shigemoto-Mogami Y. & Inoue K. Dynamic inhibition of excitatory synaptic transmission by astrocyte-derived ATP in hippocampal cultures. Proc Natl Acad Sci USA 100, 11023–11028 (2003). 12958212 10.1073/pnas.1834448100 PMC196920

[b31] Pascual O. . Astrocytic purinergic signaling coordinates synaptic networks. Science 310, 113–116 (2005). 16210541 10.1126/science.1116916

[b32] Imura Y. . Microglia release ATP by exocytosis. Glia 61, 1320–1330 (2013). 23832620 10.1002/glia.22517

[b33] Okada S. F. . Inflammation promotes airway epithelial ATP release via calcium-dependent vesicular pathways. Am J Respir Cell Mol Biol 49, 814–820 (2013). 23763446 10.1165/rcmb.2012-0493OC PMC3931099

[b34] Koizumi S. Synchronization of Ca^2+^ oscillations: involvement of ATP release in astrocytes. FEBS J 277, 286–292 (2010). 19895581 10.1111/j.1742-4658.2009.07438.x

[b35] Birder L. & Andersson K. E. Urothelial signaling. Physiol Rev 93, 653–680 (2013). 23589830 10.1152/physrev.00030.2012 PMC3768101

[b36] Birder L. A. Urothelial signaling. Auton Neurosci 153, 33–40 (2010). 19666243 10.1016/j.autneu.2009.07.005 PMC2818048

[b37] Hawthorn M. H., Chapple C. R., Cock M. & Chess-Williams R. Urothelium-derived inhibitory factor(s) influences on detrusor muscle contractility *in vitro*. Br J Pharmacol 129, 416–419 (2000). 10711338 10.1038/sj.bjp.0703068 PMC1571854

[b38] Templeman L., Chapple C. R. & Chess-Williams R. Urothelium derived inhibitory factor and cross-talk among receptors in the trigone of the bladder of the pig. J Urol 167, 742–745 (2002). 11792964 10.1016/S0022-5347(01)69137-7

[b39] Chaiyaprasithi B., Mang C. F., Kilbinger H. & Hohenfellner M. Inhibition of human detrusor contraction by a urothelium derived factor. J Urol 170, 1897–1900 (2003). 14532802 10.1097/01.ju.0000091870.51841.ae

[b40] Santoso A. G., Sonarno I. A., Arsad N. A. & Liang W. The role of the urothelium and ATP in mediating detrusor smooth muscle contractility. Urology 76, 1267 e1267–1212 (2010). 20869103 10.1016/j.urology.2010.06.040

[b41] Morris C. E. & Homann U. Cell surface area regulation and membrane tension. J Membr Biol 179, 79–102 (2001). 11220366 10.1007/s002320010040

[b42] Burnstock G., Dumsday B. & Smythe A. Atropine resistant excitation of the urinary bladder: the possibility of transmission via nerves releasing a purine nucleotide. Br J Pharmacol 44, 451–461 (1972). 4339250 10.1111/j.1476-5381.1972.tb07283.x PMC1665813

[b43] Giglio D., Delbro D. S. & Tobin G. On the functional role of muscarinic M2 receptors in cholinergic and purinergic responses in the rat urinary bladder. Eur J Pharmacol 428, 357–364 (2001). 11689195 10.1016/s0014-2999(01)01286-9

[b44] Aronsson P., Andersson M., Ericsson T. & Giglio D. Assessment and characterization of purinergic contractions and relaxations in the rat urinary bladder. Basic Clin Pharmacol Toxicol 107, 603–613 (2010). 20406212 10.1111/j.1742-7843.2010.00554.x

[b45] Sadananda P., Drake M. J., Paton J. F. & Pickering A. E. A functional analysis of the influence of beta3-adrenoceptors on the rat micturition cycle. J Pharmacol Exp Ther 347, 506–515 (2013). 24008334 10.1124/jpet.113.207340 PMC3807064

[b46] Masunaga K., Chapple C. R., McKay N. G., Yoshida M. & Sellers D. J. The beta3-adrenoceptor mediates the inhibitory effects of beta-adrenoceptor agonists via the urothelium in pig bladder dome. Neurourol Urodyn 29, 1320–1325 (2010). 20151470 10.1002/nau.20838

[b47] Kanai A. . Researching bladder afferents-determining the effects of beta(3) -adrenergic receptor agonists and botulinum toxin type-A. Neurourol Urodyn 30, 684–691 (2011). 21661014 10.1002/nau.21102

[b48] Nakagomi H. . Vesicular nucleotide transporter (VNUT) is a key molecule for mechanosensing in the human urinary bladder: negative correlation between VNUT expression in human bladder mucosa and first desire to void. Neurourol Urodyn 31, 741–742 (2012).

[b49] Wyndaele J. J. & De Wachter S. The basics behind bladder pain: a review of data on lower urinary tract sensations. Int J Urol 10 Suppl, S49–55 (2003). 14641415 10.1046/j.1442-2042.10.s1.11.x

[b50] Sakamoto S. . Impairment of vesicular ATP release affects glucose metabolism and increases insulin sensitivity. Sci Rep 4, 6689 (2014). 25331291 10.1038/srep06689 PMC4204045

[b51] Sun Y., Keay S., De Deyne P. G. & Chai T. C. Augmented stretch activated adenosine triphosphate release from bladder uroepithelial cells in patients with interstitial cystitis. J Urol 166, 1951–1956 (2001). 11586266

[b52] Yu W. Polarized ATP distribution in urothelial mucosal and serosal space is differentially regulated by stretch and ectonucleotidases. Am J Physiol Renal Physiol 309, F864–872 (2015). 26336160 10.1152/ajprenal.00175.2015 PMC4652075

[b53] Yoshiyama M. . Functional roles of TRPV1 and TRPV4 in control of lower urinary tract activity: dual analysis of behavior and reflex during the micturition cycle. Am J Physiol Renal Physiol 308, F1128–1134 (2015). 25761879 10.1152/ajprenal.00016.2015

[b54] Yoshiyama M. . Sex-related differences in activity of lower urinary tract in response to intravesical acid irritation in decerebrate unanesthetized mice. Am J Physiol Regul Integr Comp Physiol 295, R954–960 (2008). 18650315 10.1152/ajpregu.90406.2008

[b55] Apodaca G. . Disruption of bladder epithelium barrier function after spinal cord injury. Am J Physiol Renal Physiol 284, F966–976 (2003). 12527557 10.1152/ajprenal.00359.2002

[b56] Lewis S. A. & Diamond J. M. Na+ Transport by Rabbit Urinary-Bladder, a Tight Epithelium. J Membrane Biol 28, 1–40 (1976). 9512 10.1007/BF01869689

[b57] Lewis S. A. & de Moura J. L. Incorporation of cytoplasmic vesicles into apical membrane of mammalian urinary bladder epithelium. Nature 297, 685–688 (1982). 6283364 10.1038/297685a0

[b58] Truschel S. T. . Stretch-regulated exocytosis/endocytosis in bladder umbrella cells. Mol Biol Cell 13, 830–846 (2002). 11907265 10.1091/mbc.01-09-0435 PMC99602

[b59] Wang E., Truschel S. & Apodaca G. Analysis of hydrostatic pressure-induced changes in umbrella cell surface area. Methods 30, 207–217 (2003). 12798135 10.1016/s1046-2023(03)00027-6

[b60] Yoshiyama M., deGroat W. C. & Fraser M. O. Influences of external urethral sphincter relaxation induced by alpha-bungarotoxin, a neuromuscular junction blocking agent, on voiding dysfunction in the rat with spinal cord injury. Urology 55, 956–960 (2000). 10840125 10.1016/s0090-4295(00)00474-x

[b61] Yoshiyama M., Araki I., Kobayashi H., Zakoji H. & Takeda M. Functional roles of TRPV1 channels in lower urinary tract irritated by acetic acid: *in vivo* evaluations of the sex difference in decerebrate unanesthetized mice. Am J Physiol Renal Physiol 298, F1351–1359 (2010). 20237234 10.1152/ajprenal.00695.2009

