# Supplementary material for: Urothelial ATP exocytosis: regulation of bladder compliance in the urine storage phase
Source: Sci Rep. 2016 Jul 14;6:29761. doi: 10.1038/srep29761 (PMC4944198; doi:10.1038/srep29761)
Supplement: Supplementary Information [file srep29761-s1.pdf]

Supplementary Information

Urothelial ATP exocytosis: regulation of bladder compliance in the urine storage phase

Hiroshi Nakagomi[1], Mitsuharu Yoshiyama[1], Tsutomu Mochizuki[1], Tatsuya Miyamoto[1], Ryohei Komatsu[2], Yoshio Imura[2], Yosuke Morizawa[2], Miki Hiasa[3], Takaaki Miyaji[4], Satoru Kira[1], Isao Araki[5], Kayoko Fujishita[2], Keisuke Shibata[2,9], Eiji Shigetomi[2,9], Youichi Shinozaki[2,9], Reiko Ichikawa[6], Hisayuki Uneyama[6], Ken Iwatsuki[6], Masatoshi Nomura[7], William C. de Groat[8], Yoshinori Moriyama[3], Masayuki Takeda[1] and Schuichi Koizumi[*2,9]

[1]Department of Urology, Interdisciplinary Graduate School of Medicine, University of Yamanashi, Yamanashi 409-3898, Japan

[2]Department of Neuropharmacology, Interdisciplinary Graduate School of Medicine, University of Yamanashi, Yamanashi 409-3898, Japan

[3]Department of Membrane Biochemistry, Okayama University Graduate School of Medicine, Density, and Pharmaceutical Science, Okayama 700-8530, Japan

[4]Advanced Science Research Center, Okayama University, Okayama 700-8530, Japan

[5]Department of Urology, Shiga University of Medical Science, Shiga 520-2192, Japan

[6]Institute of Life Sciences, Ajinomoto Co. Inc., Kawasaki 210-8681, Japan

[7]Department of Endocrine and Metabolic Diseases/Diabetes Mellitus Kyushu University Hospital, Fukuoka 812-8582, Japan

[8]Department of Pharmacology and Chemical Biology, University of Pittsburgh, Pittsburgh, PA 15213, USA

[9]Japan Science and Technology Agency, CREST, Tokyo 102-0076, Japan

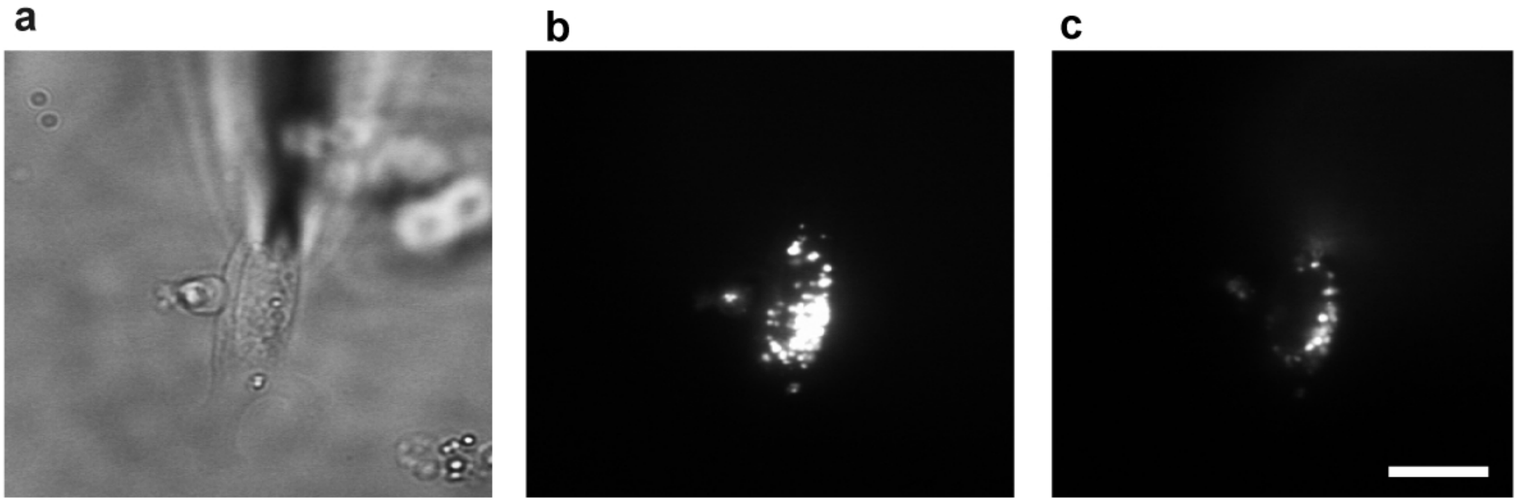

Fig. S1 Visualization of exocytosis of ATP as a decrease in staining of quinacrine in primary urothelial cells.

a. Phasecontrast image showing cells and a micropipette used for mechanical stimulation of the cells. b & c. Quinacrine-positive signals before (b) and after (c) mechanical stimulation. The intensity of staining in individual quinacrine-positive vesicles were decreased with different kinetics.

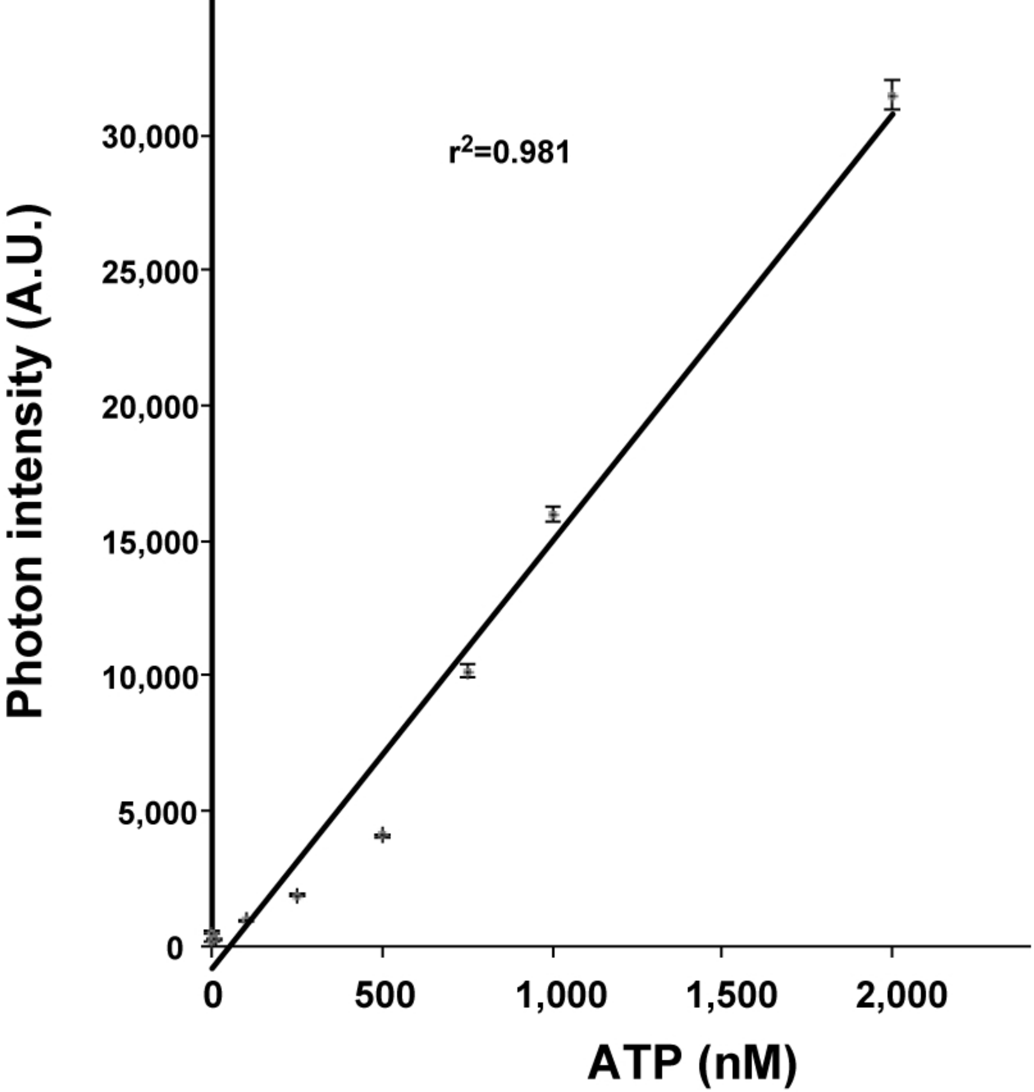

Fig. S2 Relationship between photon intensity and ATP concentration.
Standard calibration curve shows a correlation between photon intensity and ATP concentration. Data are means ± SEM (n=6).

Supplementary table 1

Comparison of body and urinary tracts weight between WT and VNUT-KO mice

	WT	VNUT-KO	*P*-value
Body weight (g)	22.78 ± 0.34 (n=5)	23.32 ± 0.68 (n=5)	0.499
Bladder weight (mg)	21.54 ± 1.02 (n=5)	21.66 ± 0.00 (n=5)	0.93
Left kidny weight (g)	0.146 ± 0.01 (n=5)	0.123 ± 0.01 (n=5)	0.164
Right kidny weight (g)	0.146 ± 0.01 (n=5)	0.141 ± 0.01 (n=5)	0.587

Values represent means ± s.e.m. for the number of animals analyzed (n). P value were derived from unpaired t-test.
